# Efficacy of Tai Chi and qigong for the prevention of stroke and stroke risk factors: A systematic review with meta-analysis

**DOI:** 10.1097/MD.0000000000008517

**Published:** 2017-11-10

**Authors:** Romy Lauche, Wenbo Peng, Caleb Ferguson, Holger Cramer, Jane Frawley, Jon Adams, David Sibbritt

**Affiliations:** a Faculty of Health, Australian Research Centre in Complementary and Integrative Medicine (ARCCIM); b Faculty of Health, Centre for Cardiovascular and Chronic Care, University of Technology Sydney, Sydney, NSW, Australia; c Department of Internal and Integrative Medicine, Kliniken Essen-Mitte, Faculty of Medicine, University of Duisburg-Essen, Essen, Germany.

**Keywords:** diabetes, dyslipidaemia, hypertension, metabolic syndrome, obesity, overweight, prevention, qigong, risk factor, stroke, Tai Chi

## Abstract

**Background:**

This review aims to summarize the evidence of Tai Chi and qigong interventions for the primary prevention of stroke, including the effects on populations with major stroke risk factors.

**Methods:**

A systematic literature search was conducted on January 16, 2017 using the PubMed, Scopus, Cochrane Library, and CINAHL databases. Randomized controlled trials examining the efficacy of Tai Chi or qigong for stroke prevention and stroke risk factors were included. Risk of bias was assessed using the Cochrane Risk of Bias tool.

**Results:**

Twenty-one trials with n = 1604 patients with hypertension, hyperlipidaemia, diabetes, overweight or obesity, or metabolic syndrome were included. No trials were found that examined the effects of Tai Chi/qigong on stroke incidence. Meta-analyses revealed significant, but not robust, benefits of Tai Chi/qigong over no interventions for hypertension (systolic blood pressure: −15.55 mm Hg (95% CI: −21.16; −9.95); diastolic blood pressure: −10.66 mm Hg (95% CI: −14.90, −6.43); the homeostatic model assessment (HOMA) index (−2.86%; 95% CI: −5.35, −0.38) and fasting blood glucose (−9.6 mg/dL; 95% CI: −17.28, −1.91), and for the body mass index compared with exercise controls (−1.65 kg/m^2^; 95% CI: −3.11, −0.20). Risk of bias was unclear or high for the majority of trials and domains, and heterogeneity between trials was high. Only 6 trials adequately reported safety. No recommendation for the use of Tai Chi/qigong for the prevention of stroke can be given.

**Conclusion:**

Although Tai Chi and qigong show some potential more robust studies are required to provide conclusive evidence on the efficacy and safety of Tai Chi and qigong for reducing major stroke risk factors.

## 1 Introduction

Stroke is one of the leading causes of mortality and disability worldwide^[1]^ and together with ischemic heart disease (IHD) it was responsible for nearly 1 in 4 deaths in 2010.^[2]^ Mortality rates due to stroke have been on an overall decline over recent decades, both as a result of the drop in stroke incidence and lower fatality rates. However 6.7 million people worldwide died from stroke in 2012,^[3]^ and the importance of preventive measures is highlighted by the growing proportion of stroke survivors who are between the ages of 20 and 64.^[4]^

Multiple complex risk factors contribute to a stroke incidence and while some factors such as age, gender, ethnicity, or heredity are nonmodifiable, the majority of risk factors are lifestyle related and largely modifiable.^[5]^ A recent study indicated that 10 potentially modifiable risk factors are collectively associated with 90% of the population attributable risk,^[6]^ with the major risk factor being hypertension, followed by factors such as hyperlipidaemia, diabetes, unhealthy diets, overweight and/or obesity, tobacco use, excessive alcohol consumption, illicit drug use, and a lack of physical activity.^[1,7]^ According to a Centers for Disease Control and Prevention (CDC) study, 1 in 2 US adults were found to have hypertension on blood pressure measurements; however, one-third of those did not receive an appropriate diagnosis or medication,^[8]^ highlighting the need for greater treatment and evaluation of blood pressure lowering interventions.

Controlling modifiable risk factors is the key to decreasing the risk of stroke and exercise if one of the most frequently recommended interventions, due to an association with reduction in body weight,^[9]^ blood pressure,^[10,11]^ and triglycerides^[12]^ as well as glucose regulation.^[13]^ Conventional exercise interventions usually include aerobic, strength, or flexibility training; however, alternative exercise interventions such as Tai Chi and qigong have recently gained popularity in the general population for disease prevention.^[14]^

Tai Chi is a mind-body exercise originating in China. It incorporates slow dance-like movements, and integrates musculoskeletal, breathing, and meditation training. Tai Chi is often used for health purposes^[14]^ and a growing body of evidence supports Tai Chi's potential efficacy and safety for a variety of health conditions such as cardiovascular diseases,^[15,16]^ balance and neuromuscular conditions,^[17–19]^ cognition, and psychological well-being.^[20,21]^ In the United States research has led to the endorsement of Tai Chi for maintaining health and supporting rehabilitation by national organizations such as the CDC.^[22]^ Qigong, another mind-body practice originating in China, shares many of Tai Chi's principles; however qigong movements are typically limited to more simplistic and repetitive choreographed routines. Tai Chi (and qigong) can both easily be adapted for the needs of its users, and Tai Chi has been found to be safe for a wide variety of users including older people with chronic health conditions.^[23]^

Since stroke incidence increases with advanced age, the use of Tai Chi and qigong may constitute a viable intervention not only to improve balance and agility in the elderly, but also to support stroke prevention. However, with regards to the latter no synthesis of evidence examining the effects of Tai Chi or qigong for stroke prevention and/or reduction of major risk factors is currently available.

Aim: To examine and summarize the evidence regarding Tai Chi and qigong interventions for the primary prevention of stroke, including the effects of such intervention on populations with selected major stroke risk factors.

## 2 Materials and methods

This study is a systematic review of published studies; as such ethical approval was not required. The systematic review was registered in the PROSPERO database (#CRD42017056307). A protocol was developed using the Preferred Reporting Items for Systematic Review and Meta-Analysis Protocols (PRISMA-P) 2015 Statement.^[24]^ The following risk factors for stroke were identified and considered of high relevance by the authors and stroke expert following consultations: hypertension, hypercholesterinemia, diabetes, overweight and obesity, and combinations of these symptoms (metabolic syndrome), tobacco, and alcohol use.

### 2.1 Search strategy

A database search was conducted on January 16, 2017 to identify original research investigating the effects of Tai Chi and qigong on stroke incidence, and effects on patients with selected stroke risk factors, published between 1996 (when the first CONSORT statement was published)^[25]^ and 2016. The time limit was chosen to ensure that (hypothetically) all authors of potential articles had been able to follow the guideline for reporting their clinical trials, supporting a comprehensive risk of bias assessment. The search included the following databases: Scopus, the Cochrane Library, PubMed/Medline, and CINAHL. The search terms employed were constructed around search terms for Tai Chi or qigong, and terms for stroke or the following risk factors hypertension, hypercholesterinemia, diabetes, overweight and obesity, and tobacco and alcohol use. The complete search strategy for PubMed can be found in Table 1. Reference lists of published review articles were also reviewed to ensure all relevant known articles were included.

**Table 1:**
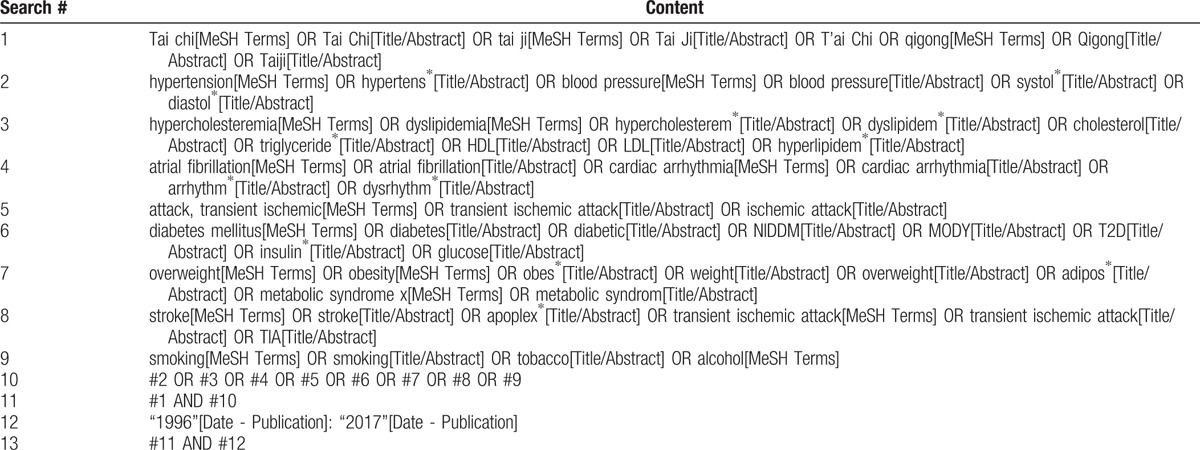
Complete search strategy for the PubMed database.

### 2.2 Eligibility criteria

Papers reporting findings from randomized controlled trials (RCTs) on adults with or without risk factors who were monitored for stroke incidence; or patients who fulfilled the criteria for one of the stroke risk factors were included irrespective of gender.

Eligible studies were those that examined the therapeutic effect of Tai Chi or qigong regardless of the form, tradition, frequency, and duration of the intervention. To meet the inclusion criteria manuscripts had to report on studies that compared Tai Chi or qigong to no treatment, nonexercise control intervention and exercise control interventions.

Manuscripts were included if they reported upon research which measured stroke incidence and/or relevant outcomes for the respective risk factors, including: blood pressure for hypertension; triglycerides, and total and low-density lipoproteins (LDL)/high-density lipoproteins (HDL) cholesterol for hypercholesterinemia; weight, body mass index, waist/hip circumference, and body fat percentage for overweight/obesity; fasting or postprandial blood glucose, insulin, HbA1c and the homeostatic model assessment (HOMA) index for diabetes; number of participants successfully quitting tobacco or alcohol use in trials for health behavior changes; and safety for all trials. For the purpose of this review, only articles published in English and German were included.

### 2.3 Review of records

All articles were imported into Endnote (Version X8, Clarivate Analytics). Two reviewers (RL, WP) independently screened abstracts of records and full texts of potentially eligible articles were retrieved. Full texts were read by 2 authors (RL, WP) and the final list of eligible studies was compiled. If discrepancies between the 2 reviewers occurred those items were discussed with a third reviewer (CF) to achieve a consensus.

### 2.4 Data extraction

Two pairs of trained researchers (RL, WP; RL, JCA) extracted data independently using an a priori data extraction form, including country of origin, sample characteristics (sample size, age, gender, ethnicity and inclusion criteria), intervention data (treatment and control group) and outcome measures (dependent variables, measurement time points). If discrepancies between the 2 reviewers occurred those items were discussed with a third reviewer (CF) to achieve a consensus.

### 2.5 Risk of bias

Risk of bias was assessed using the Cochrane Risk of Bias Tool.^[26]^ Within each of the domains, the risk of bias was assessed as low; unclear or high separately by 2 reviewers (RL, WP). A third reviewer (CF) was consulted when a difference of opinion arose, until consensus was achieved.

### 2.6 Data synthesis

The overall effect sizes for each outcome were determined using meta-analytic approaches if at least 2 studies assessed the specific outcome. The Review Manager 5 software (Version 5.3, The Nordic Cochrane Centre Copenhagen) was used, and random effects models were applied. Separate meta-analyses were conducted to examine the effects of Tai Chi and qigong compared with: pooled exercise control interventions; and pooled nonexercise control interventions. Mean differences (MD) with 95% confidence intervals (CI) were reported. Attempts were made to obtain missing data from the studies’ authors by email. Negative MDs (i.e., lower values in the Tai Chi/qigong groups) were defined to indicate benefits of Tai Chi and qigong over the control intervention for all outcomes except for HDL cholesterol where a negative MD (i.e., lower values in the Tai Chi/gigong group) was defined to indicate benefits of the control interventions over Tai Chi/qigong.

Statistical heterogeneity between the studies was determined using I^2^ statistics, and the magnitude of heterogeneity was categorized as I^2^ = 0% to 24%: low; I^2^ = 25% to 49%: moderate; I^2^ = 50% to 74%: substantial; and I^2^ = 75% to 100%: considerable heterogeneity;^[26,27]^ and the *χ*^2^ test was used to assess the statistical significance of heterogeneity between trials. In the face of the low power of this test in small samples, a *P* value ≤.10 was regarded to indicate significant heterogeneity.^[28]^

To test the robustness of significant results, sensitivity analyses were conducted for studies with high versus low risk of bias in the following domains: selection bias (random sequence generation and allocation concealment); detection bias (blinding of outcome assessment); and attrition bias (incomplete outcome data).

## 3 Results

**Figure 1:**
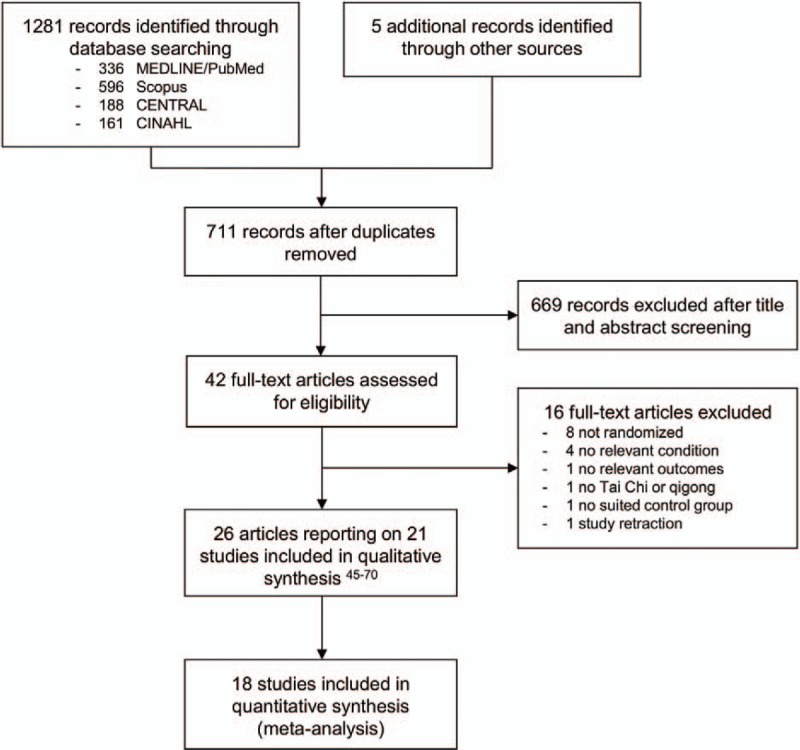
Flowchart of literature search and study selection.

A flowchart of literature search and study selection is presented in Fig. 1. Overall 1281 records were identified during database searches and another 5 records during manual search. After removing duplicates, a pool of 711 records remained, of which 42 were assessed as full-text articles. Of these, 16 had to be excluded because they included patients without hypertension,^[29–31]^ with elevated glucose instead of diabetes only,^[32]^ reported no relevant outcomes,^[33]^ were not randomized,^[34–41]^ did not use Tai Chi or qigong,^[42]^ or used Tai Chi or qigong in both arms,^[43]^ or the study was retracted.^[44]^ A final total of 26 publications were included in the systematic review, reporting a total of 21 trials and n = 1604 participants.^[45–70]^ Of those, 8 trials investigated the effects of Tai Chi or qigong on hypertension,^[46,48,49,54–56,60,63,67]^ 1 on hyperlipidaemia,^[61]^ 7 on diabetes,^[47,53,58,59,62,65,66,68–70]^ 6 on overweight and obesity,^[45,47,50–52,57,61]^ and 1 on the metabolic syndrome.^[64]^ No included trials were identified which examined the effects of Tai Chi or qigong on tobacco or alcohol use. All included studies except 1 German study^[60]^ were published in English. The majority of studies which met the inclusion criteria originated in Asia, including China (including Hong Kong and Taiwan),^[47,49,61,63,67,68,70]^ Malaysia,^[46,48]^ Korea,^[54–56]^ and Thailand;^[64,69]^ Europe, including France^[50]^ and the United Kingdom;^[60]^ the United States;^[45,47,51,52,59,62]^ and Australia.^[53,57,58,65,66]^ Below we discuss the studies included and the results of this review separately for each risk factor population. Characteristics of the included studies can be found in Table 2  .

**Table 2:**
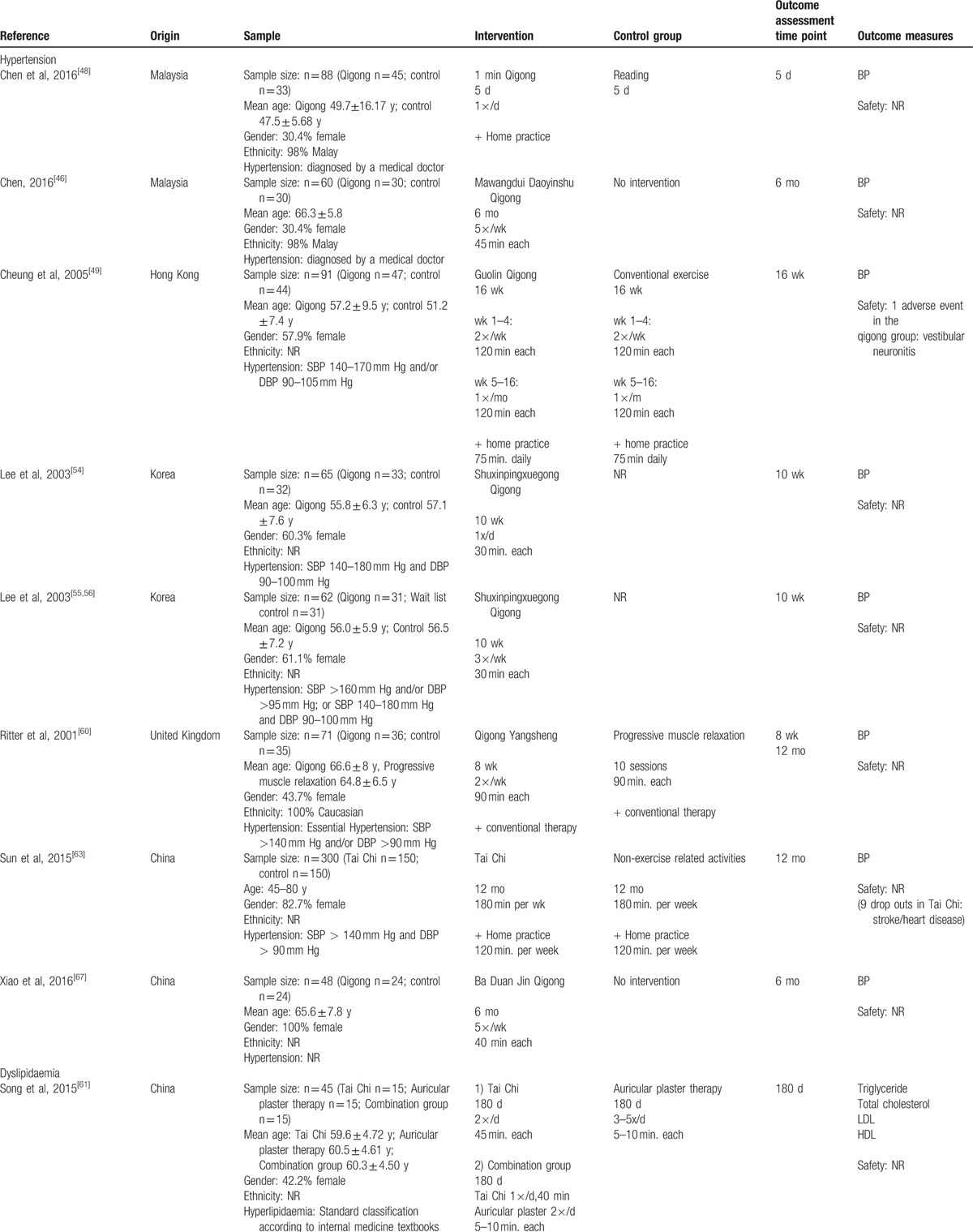
Characteristics of studies included in this review.

**Table 2 (Continued):**
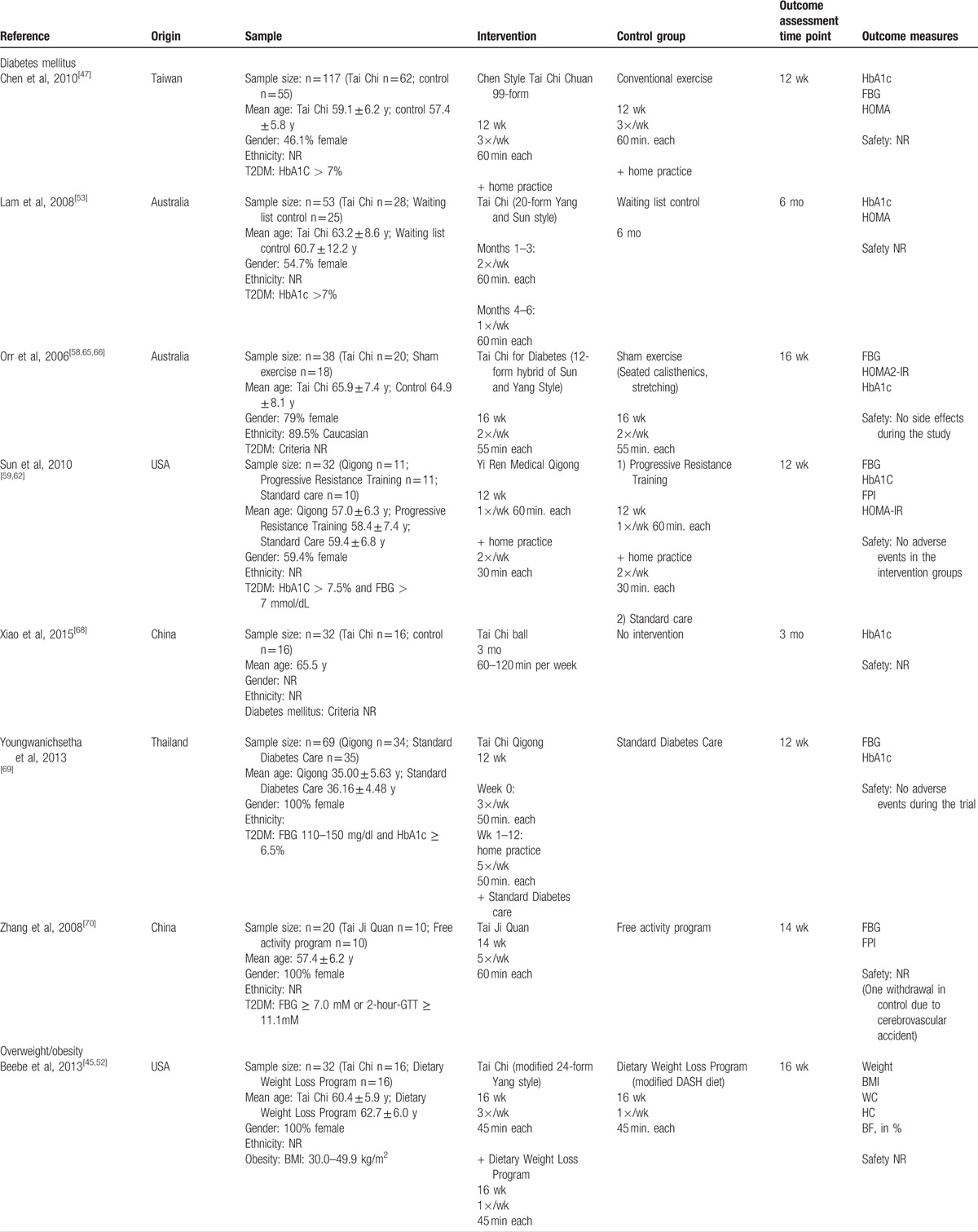
Characteristics of studies included in this review.

**Table 2 (Continued):**
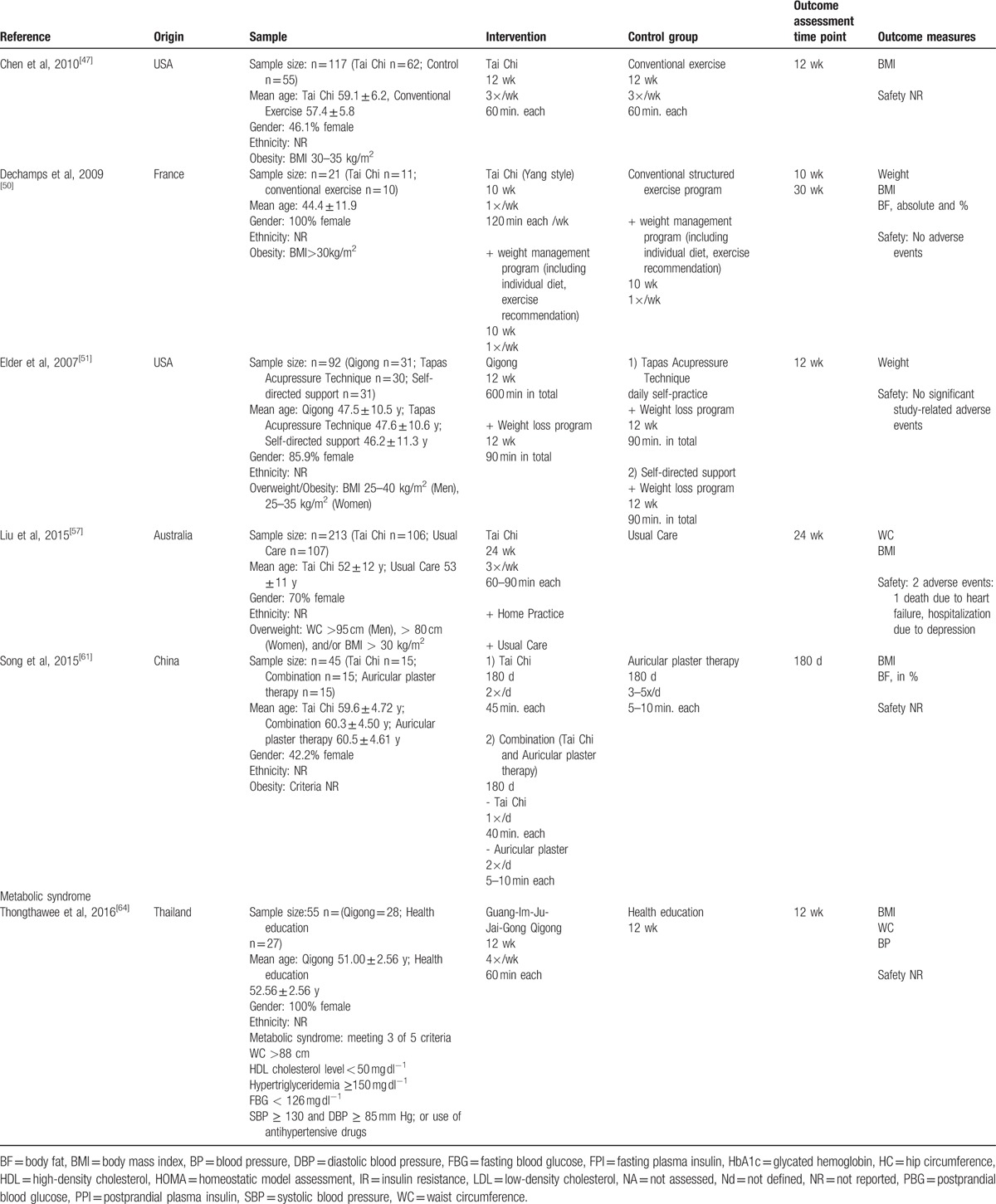
Characteristics of studies included in this review.

### 3.1 Stroke prevention

The literature search revealed no trial examining the effects of Tai Chi/qigong on the incidence of stroke or transient ischemic attack.

### 3.2 Hypertension

A total of 8 trials with 785 patients examined hypertension. Of these 1 trial did not report the diagnostic criteria,^[67]^ while 2 trials only stated that patients were diagnosed by a medical doctor.^[46,48]^ The other 5 trials examining hypertension included patients with a systolic blood pressure above 140 mm Hg, and/or a diastolic blood pressure above 90 mm Hg, with some trials also having an upper blood pressure limit for inclusion. Seven of the trials examining hypertension employed qigong and 1 Tai Chi as an intervention.^[63]^ The trials examining hypertension reported control groups which received no intervention (n = 4);^[46,54–56,67]^ undertook exercises^[49]^ or nonexercise related activities (n = 3)^[48,60,62]^ such as progressive muscle relaxation^[60]^ or reading.^[48]^ The duration of the interventions reported ranged from 5 days to 12 months, with the majority of trials conducted between 8 and 24 weeks (median 12 weeks). The frequency of the Tai Chi or qigong interventions ranged from 2 days a week to daily (median 4 times per week), and 3 trials explicitly included self-directed home practice.

**Figure 2:**
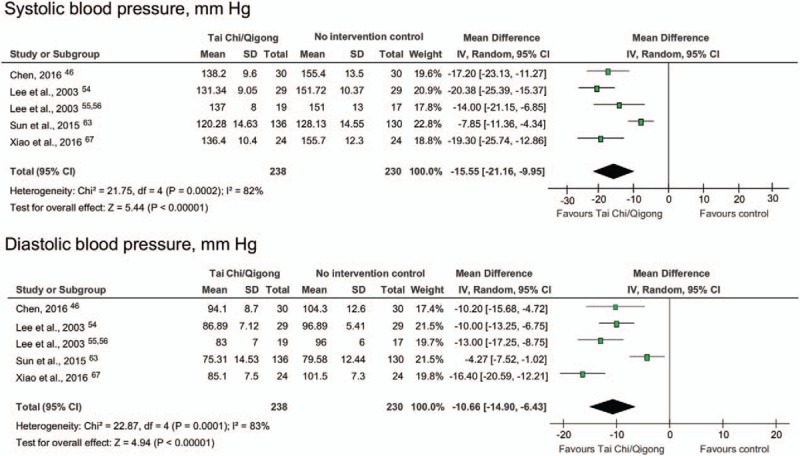
Forest plot and effect sizes for Tai Chi/qigong compared with no intervention controls and exercise controls for systolic and diastolic blood pressure, in mm Hg.

A meta-analysis was conducted for the effects of Tai Chi/qigong on blood pressure compared with a pooled group that included no intervention controls, or interventions such as reading or computer training (see Fig. 2, and Table 3). Results of pooling 5 studies with 468 participants showed significant benefits of Tai Chi/qigong over no intervention with mean group difference in systolic blood pressure of −15.55 mm Hg (95% CI −21.16; −9.95; I^2^ = 82%); and mean group difference in diastolic blood pressure of −10.66 mm Hg (95% CI: −14.90, −6.43; I^2^ = 83%). Stepwise exclusions of trials revealed that the high level of heterogeneity was a result of different effect sizes between Sun and Buys^[63]^ and the remaining 4 studies.^[46,54–56,67]^ A trial that compared qigong with Progressive Muscle Relaxation found no group differences for blood pressure.^[60]^ The only trial that compared qigong with conventional exercise found no group differences for blood pressure, and concluded that both interventions had similar moderate effects.^[49]^

**Table 3:**
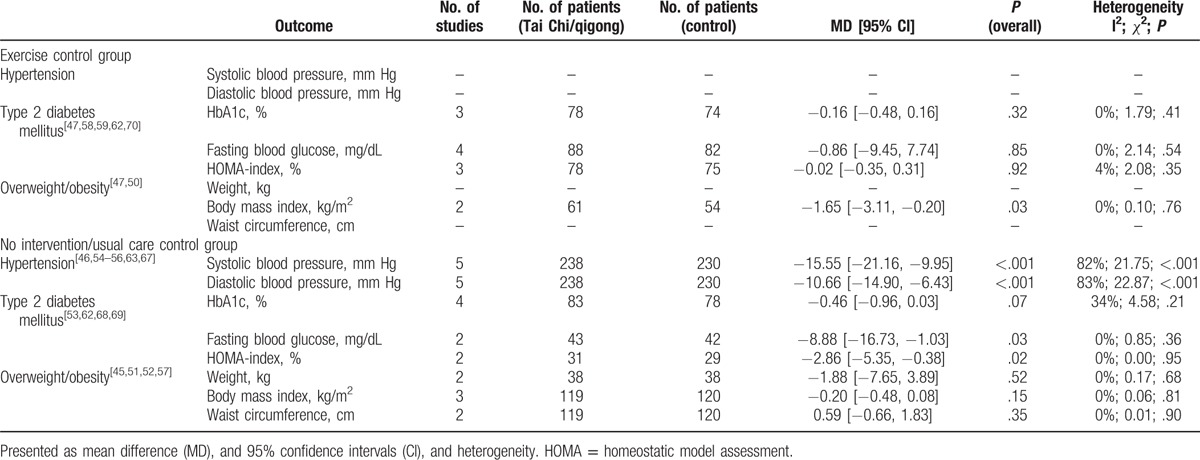
Effects of meta-analyses for the comparison of Tai Chi or qigong versus nonexercise and exercise control for selected outcomes.

**Figure 3:**
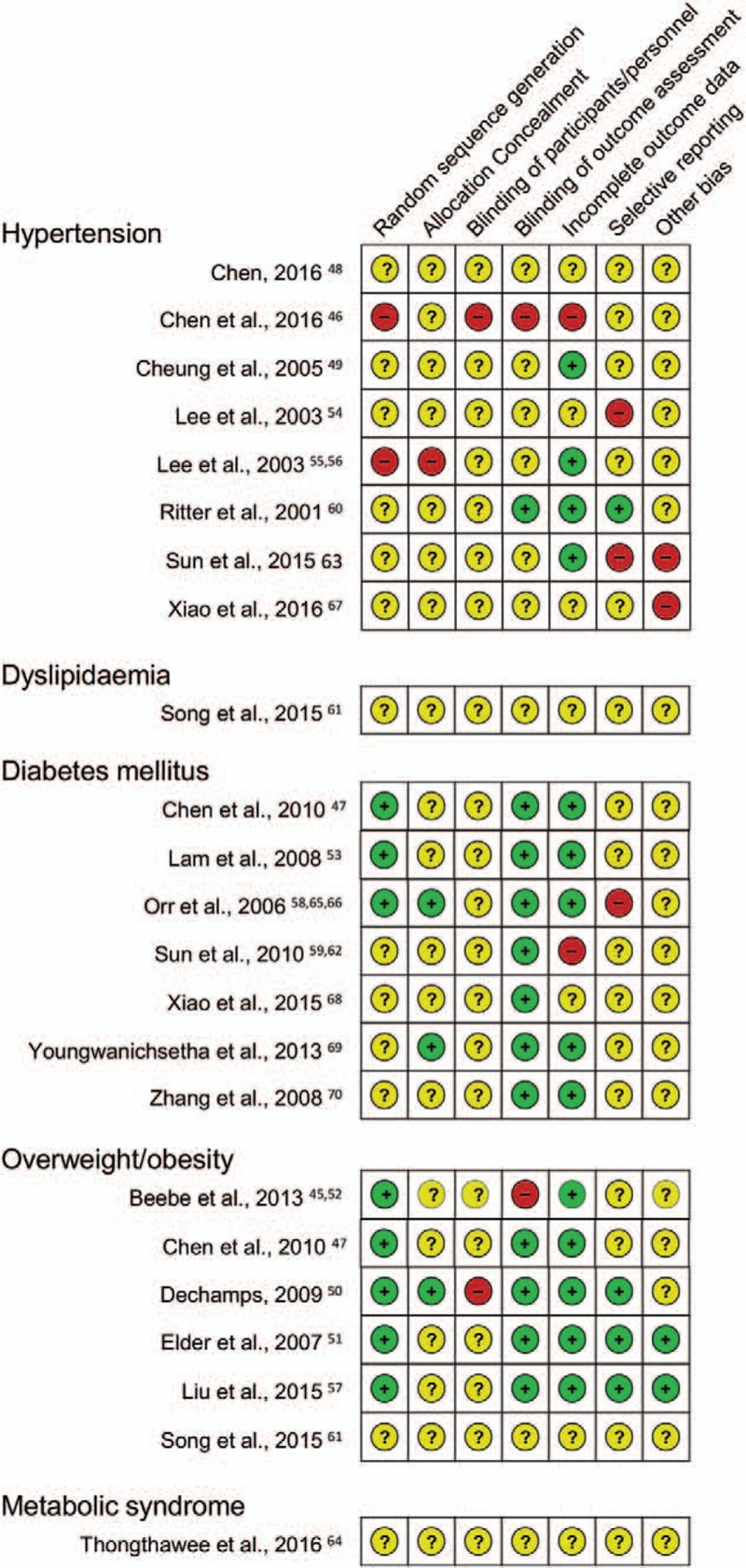
Results of the risk of bias assessment. + indicates low risk, − indicates high risk, and ? indicates unclear risk of bias.

As for risk of bias (Fig. 3), no trial reported adequate random sequence generation and allocation concealment, while 2 trials were found to have a high risk of bias regarding those 2 domains. Furthermore, only 1 trial had low risk for blinding of outcome assessment.^[60]^ As such no sensitivity analysis could be undertaken.

### 3.3 Hyperlipidaemia

Only 1 trial investigating the effects of Tai Chi on hyperlipidaemia was identified.^[61]^ The trial included 45 patients with hyperlipidaemia according to the “standard classification as per internal medicine textbooks,” and assigned them to: Tai Chi; auricular plaster therapy; or a combination of both. After 6 months of intervention, with nearly daily practice of 5 to 10 minutes, the combination group had significantly lower levels of triglycerides, and LDL cholesterol, and higher levels of HDL cholesterol compared with both Tai Chi and the auricular plaster group (*P* < .05). Due to insufficient reporting no low risk of bias could be provided for any of the domains (Fig. 3). The trial also failed to report safety-related data.

### 3.4 Diabetes

A total of 7 trials with 361 patients examined the effects of Tai Chi/qigong on diabetes. Of these, 6 specifically included patients who were diagnosed with type 2 diabetes mellitus, while 1 trial did not specify the diabetes type.^[68]^ Five trials defined specific inclusion criteria based on HbA1c levels (n = 4),^[47,53,59,62,69]^ fasting blood glucose levels (n = 3),^[59,62,69,70]^ and/or glucose tolerance test outcomes.^[70]^ Six of the trials were using Tai Chi,^[47,53,58,65,66,68,70]^ and 1 each qigong^[59,62]^ and Tai Chi qigong^[69]^ as interventions; and in 1 trial the Tai Chi interventions was specifically designed for the treatment of diabetes.^[58,65,66]^ The control groups included no intervention/standard care (n = 4);^[53,59,62,68,69]^ a non-exercise-related free activity program;^[70]^ and conventional exercise,^[47]^ sham exercise (calisthenics and gentle stretching)^[58,65,66]^ or progressive resistance exercises.^[59,62]^ The duration of the interventions reported ranged from 6 weeks to 6 months, with the majority of trials conducted between 12 and 16 weeks (median 12 weeks). The frequency of the Tai Chi or qigong interventions ranged from 1 day a week to 5 times (median 2 times per week), and 3 trials prescribed additional home practice.

A meta-analysis was conducted for the effects of Tai Chi/qigong on HbA1C, HOMA index, and fasting blood glucose compared with nonexercise control interventions and exercise control interventions (see Table 3). Results showed significant benefits of Tai Chi/qigong over no intervention/usual care (for diabetes) control intervention for fasting blood glucose (n = 2 studies with 85 participants; mean group difference: −8.88 mg/dL (95% CI: –16.73, −1.03), I^2^ = 0%), and HOMA (n = 2 studies with 60 participants; mean group difference: −2.86% (95% CI: –5.35, −0.38), I^2^ = 0), however not for HbA1C (n = 4 studies with 161 participants; mean group difference: −0.46% (95% CI: −0.96, 0.03)). No differences were found for the comparison of Tai Chi/qigong versus exercise control interventions for HbA1C (n = 3 studies with 152 participants; mean group difference: −0.16% (95% CI: –16.73, −1.03)), HOMA (n = 3 studies with 153 participants; mean group difference: −0.02% (95% CI: −0.48; 0.16)) and fasting blood glucose (n = 4 studies with 170 participants; mean group difference: −0.86 mg/dL (95% CI: –9.45 7.74)).

As for risk of bias (Fig. 3), 3 of the 7 trials examining diabetes had low risk of bias for random sequence generation,^[47,53,58,65,66]^ yet only 1 of those with low risk regarding this domain also had low risk of allocation concealment.^[58,65,66]^ Blinding of outcome assessors was mainly of low risk due to blood samples being robust against nonblinded nurses and laboratory personnel. Due to the low number of low-risk trials no sensitivity analysis could be conducted, and the effects on HOMA and fasting blood glucose could not be considered robust against potential bias.

### 3.5 Overweight and obesity

Six trials with 520 participants investigating the effects of Tai Chi/qigong on overweight and obesity were identified.^[45,47,50–52,57,61]^ Of these, 4 trials exclusively included patients with obesity,^[45,47,50,52,61]^ and 3 provided specific inclusion criteria based on the BMI. The other 2 trials included not only included obese patients but also overweight patients,^[51,57]^ based on participants’ BMI,^[51,57]^ and/or the waist circumference.^[57]^ All but 1 trial^[51]^ used Tai Chi as the intervention. Control interventions among these trails examining overweight and obesity included usual care;^[57]^ exercise,^[47,50]^ a dietary program,^[45]^ acupuncture,^[51]^ self-directed support,^[51]^ and auricular plaster therapy.^[61]^ Two of the trials examining overweight and obesity tested the effects of Tai Chi/qigong in addition to a dietary weight loss program compared with the weight loss program alone,^[45,50]^ and 1 of the trials compared Tai Chi/qigong in addition with a dietary weight loss program to the weight loss program combined with conventional exercise.^[51]^ The duration of the interventions ranged from 10 to 30 weeks (median 14 weeks), and the frequency of the interventions ranged from 1 to 3 days a week (median 3 times per week).

A meta-analysis was conducted for the effects of Tai Chi/qigong on weight, body mass index, and waist circumference compared with no intervention/usual care (for overweight) controls interventions and exercise control interventions (see Table 3). The analysis identified a significant benefit of Tai Chi/qigong over exercise on BMI only (n = 2 studies with 239 participants; mean difference −1.65 kg/m^2^ (95% CI: −3.11, −0.20); I^2^ = 0%). No other differences were found between Tai Chi/qigong and any of the control groups (see Table 3).

As for risk of bias (Fig. 3) all but 1 trial examining overweight and obesity^[61]^ had low risk of random sequence generation, but only 1 reported adequate allocation concealment.^[50]^ Blinded outcome assessors were used in only 3 trials.^[50,51,57]^ After excluding the unclear and high risk of bias trials, the effect on the BMI was no longer significant.

### 3.6 Metabolic syndrome

One trial was found that examined the effects of qigong for the metabolic syndrome,^[64]^ a clustering of at least 3 of the 5 risk factors hypertension, hypercholesterinemia, diabetes, obesity, and low levels of HDL cholesterol. Overall 55 female patients aged 40 to 65 years were included, and 12 weeks of qigong (4 days per week) compared with a health education program were delivered. Outcome measures employed in this study included the BMI, waist circumference and blood pressure, safety was not reported. The trial found significantly lower systolic blood pressure and smaller waist circumference after the qigong intervention compared with the education intervention. Risk of bias was unclear for all domains due to insufficient reporting in this trial (Fig. 3).^[64]^

### 3.7 Tobacco and alcohol use

No trials testing the effects of Tai Chi/qigong for patients who wanted to quit smoking or abstain from alcohol consumption were identified.

### 3.8 Safety

Of the included trials, 17 (70.8%) did not report any safety-related data. In 2 of these studies adverse events had occurred, as outlined in the studies’ flowcharts.^[61,70]^ Five out of the 21 studies reported adverse events,^[49,50,57,59,62,69]^ and 1 study each reported side effects,^[65,66]^ or significant adverse events.^[51]^ Adverse events included 1 fatality due to heart failure,^[57]^ 1 hospitalization due to depression,^[57]^ and 1 case of vestibular neuronitis,^[49]^ 1 withdrawal due to cerebrovascular accident,^[18]^ an unknown number of withdrawals due to stroke/heart disease.^[63]^ Since the reporting of those adverse events was insufficient, no information could be extracted as to whether these events were caused by the intervention.

## 4 Discussion

This systematic review has several important findings. First, no trial has yet examined the effects of Tai Chi/qigong specifically on the primary prevention of stroke. One study protocol however has been recently published to examine Tai Chi's protective effects against ischemic stroke risk in a population with an increased risk for ischemic stroke.^[71]^ While prevention trials like this may face certain challenges regarding the length of observation, and the number needed to treat (NNT, i.e., the average number of patients who need to be treated to prevent 1 additional stroke) to identify significant group differences for stroke,^[72]^ indirect evidence may be gathered from other trials examining existing evidence on the effects of Tai Chi/qigong on stroke risk factors as done by this systematic review.

### 4.1 Hypertension

This systematic review found that Tai Chi/qigong may significantly reduce blood pressure, with average reductions of 15.55 mm Hg systolic and 10.66 mm Hg diastolic blood pressure when compared with no intervention. The magnitude of these improvements is large, and appears to be clinically relevant (at least 5–10 mm Hg reduction), which is in line with the results found in a previous systematic review on Tai Chi for essential hypertension.^[73]^ None of the trials included in the present meta-analyses however had a low risk of bias regarding random sequence generation, random allocation concealment and blinding of outcome assessors, and together with the high level of heterogeneity between trials the effects of Tai Chi/qigong on hypertension compared with no treatment cannot be considered robust against risk of bias.

Interestingly, the systematic reviews (present and prior) indicate that the effects of Tai Chi/gigong on blood pressure might be larger than those reported for aerobic exercise with reductions of 7 mm Hg systolic and 5 mm Hg diastolic blood pressure for the latter.^[74]^ However, the confidence intervals of the effects found for Tai Chi/qigong are overlapping with the effects found for aerobic exercise, and as such they may not necessarily represent significant differences between the different types of exercise. Furthermore due to a potential risk of bias in those trials the effects of Tai Chi/qigong on hypertension compared with exercise controls cannot be considered robust.

Future studies on Tai Chi/qigong for hypertension should ensure rigorous methodology and reporting to strengthen the validity of results. Such proposed research should further include measures of responders, including rates of participants who had successfully lowered their blood pressure below the target of 140 mm Hg systolic and/or 90 mm Hg diastolic, and should include a detailed description and analysis of concomitant medication use. Since the majority of trials lacked such information, no recommendation for the use of Tai Chi/qigong to treat hypertension can be made. Nevertheless Tai Chi/qigong are considered safe in general,^[23]^ and they might be considered for adults who are unwilling to use conventional exercises including patients undergoing cardiac rehabilitation,^[75]^ or older adults who are fragile and at elevated risk of falling, as Tai Chi has been shown to be effective as a fall prevention intervention.^[17–19,23]^

### 4.2 Diabetes

The present review also found limited effects of Tai Chi/qigong for fasting blood glucose, and HOMA in patients with type 2 diabetes mellitus, at least when compared with no intervention/usual care (for diabetes) controls. Only very few trials had an overall low risk of bias and the majority of trials reviewed lacked comprehensive and detailed description and analysis of medication and concomitant interventions including exercise and nutrition. Physical activity and medical nutrition advice or therapy are standard recommendations in type 2 diabetes mellitus management guidelines,^[76,77]^ and it can be assumed that medication and lifestyle changes themselves may have a huge impact on diabetes outcomes. As such, the insufficient reporting of concomitant interventions together with the potential risk of bias in those trials the evidence found in this review cannot be considered conclusive as has been concluded by a prior recent review as well.^[78]^ Despite these circumstances, Tai Chi has nevertheless been recommended by the American Diabetes Association to improve muscular strength and balance.^[76]^

### 4.3 Weight

With regard to weight management, our review shows only small effects have been found for the reduction in BMI after Tai Chi/qigong intervention, when compared with exercise interventions, but not compared with no intervention/usual care (for overweight) controls. It seems somewhat surprising to find an effect of Tai Chi/qigong compared with exercise controls while no effects have been found compared with intervention/usual care controls. However the sample size for each comparison was small; and despite the risk of bias for random sequence generation was low for the majority of trials, the risk of bias for allocation concealment and blinding of participants and outcome assessors was not low in general. As such, no conclusive judgement can be made regarding the efficacy of Tai Chi/qigong for weight loss based on the findings of this systematic review, and since no prior systematic review exists, our results cannot be compared to existing evidence synthesis.

### 4.4 Safety

Considering that two-thirds of included trials did not report safety data, future trials need to ensure comprehensive and rigorous reporting of all adverse events. Some of the included trials also reported safety-related data insufficiently by limiting the reporting to side effects or “significant adverse event.” In accordance with Good Clinical Practice trials should report any untoward medical occurrence; that is, any abnormal laboratory finding, symptom, or disease temporally associated with study intervention, whether or not caused by the intervention.^[79]^ Those included trials that reported safety-related data, mainly included minor events, however in 1 trial several withdrawals from the study were reported due to stroke and heart attacks.

Overall, Tai Chi has been considered a low-risk intervention^[23]^ more data is desired for conclusive judgment, especially for high-risk populations.

### 4.5 Strengths and limitations

Results from this systematic review have to be interpreted with some caution as several factors limit the significance of this review. Due to the language restriction to English and German a significant number of trials may have been excluded. Given that Tai Chi/qigong are techniques originating in China this may have significantly influenced the outcome of this review. The paucity of data for example did not allow for comprehensive meta-analyses and sensitivity analyses. The heterogeneity of trials regarding included participants, intervention characteristics, durations, and control groups also limited the validity of results. Finally, the overall unclear risk of bias of the included studies did not allow for conclusive judgement of the effects of Tai Chi/qigong for stroke prevention and risk factors.

### 4.6 Implications for future trials

Further investigation and studies are desired to enhance the understanding and scientific evidence of the efficacy, safety, and mechanisms of Tai Chi and qigong for healthy people and populations at risk for stroke. Especially trials examining the benefit of Tai Chi for primary prevention of stroke in healthy adults as well as adults with high risk for stroke are urgently needed. When conducting trials, researchers need to be aware of challenges inherent to Tai Chi as a multimodal intervention^[80,81]^ with a cultural heritage, and sufficiently report the intervention characteristics. Future trials also need to ensure high methodological quality and minimize potential sources of bias by ensuring adequate randomization procedures, ensure blinding of outcome assessors, develop methods to account for nonblinding of participants, and improve general reporting and reporting of outcomes, including safety. While this will not necessarily reduce the heterogeneity between trials due to sample characteristics, settings, or interventions, it will likely reduce heterogeneity due to methodological issues.

Lastly trials should be registered in public clinical trial registries to prevent selective reporting of outcomes and results. As such, journals publishing trials on Tai Chi and qigong should make prospective trial registration mandatory.

### 4.7 Implications for practice

Even though no conclusive judgment on the effects of Tai Chi/qigong for the prevention of stroke can be made based on the results of this systematic review, the use of Tai Chi/qigong should not be discouraged at the current time unless clinically contraindicated. Tai Chi appears to have a low-risk profile, as such people with a personal preference toward Tai Chi or qigong, or people who are fragile or at risk of falls, might benefit from engaging in these forms of exercise regularly.

## 5 Conclusion

No recommendation for or against the use of Tai Chi or qigong for the primary prevention of stroke can be given at the current time. However, Tai Chi and qigong show some potential in reducing some major stroke risk factors, and as such more high-quality studies are required for conclusive judgement on the efficacy and safety of Tai Chi and qigong for healthy populations and risk factor patient samples.

## Acknowledgments

The authors wish to thank the Nancy and Vic Allen Stroke Prevention Fund for grant funding this systematic review, and Josephine C. Agu (JCA) for supporting literature management and data extraction.
